# Adolescent male with anorexia nervosa: a case report from Iraq

**DOI:** 10.1186/1753-2000-6-5

**Published:** 2012-01-26

**Authors:** Maha S Younis, Lava D Ali

**Affiliations:** 1 Department of Psychiatry, College of Medicine, Baghdad University, Iraq; 2 Suliamania University Hospital, Kurdistan region, Iraq

**Keywords:** Anorexia Nervosa, Iraq, Case report

## Abstract

This is the first reported case of an adolescent male with anorexia nervosa in Iraq. This disorder is believed to be rare in males across cultures and uncommon for both genders in Arab countries. The patient met the DSM-IV diagnostic criteria for anorexia nervosa. He was hospitalized and received medical and psychiatric treatment at local facilities as discussed below and responded well to treatment.

## Background

Anorexia nervosa (AN) is a serious eating disorder with an incidence rate of 135.7 per 100,000 per year in Western countries [1,2] characterized by self-induced starvation in which the body mass index (BMI) is below 17.5. It is associated with an array of signs and symptoms leading to serious medical complications and sometimes death [1-3]. This disorder is characterized by intentionally starving oneself and failing to maintain a minimum body weight that is considered healthy for the person's age and height. The starvation is inflicted by severe calorie restriction and/or purging behavior (self-induced vomiting, use of laxatives, enemas and diuretics). It is believed to be caused by intense fear of gaining weight or becoming fat and is associated with a disturbed body image [1-3].

AN is common in adolescents, with variable data reported about its incidence, psychopathology and outcome. It is often under-diagnosed in males because of the atypical symptoms and rarity of the condition. However, recent studies have demonstrated an increased incidence with remarkable similarities of presentation and psychopathology to that of females, except for amenorrhea [4-6].

Many Arab societies do not consider being overweight a stigma, but instead a sign of good health [7,8]. Thus, anorexia nervosa is scantily documented in the Arab world [7-9] except for a study by Al-Awadi [10]. Findings revealed that 10.9% of Omani males had disturbed eating attitudes. However, there are no population-based prevalence surveys of AN in the Arab world, and all available published reports agree on its rare incidence [11-15]. The case reported here shows the awareness of being overweight that was sensed at the age of 14, prompted by the harsh comments of the patient's friends. This case reflects anorectic symptoms and signs at an early age, possible due to the athletic ambition of boys in their early teens who want to build a more muscular body [4].

## Case presentation

MR was a 14-year-old Muslim Kurdish student living in Sulimania, northeast of Iraq. His parents were of an educated middle class family who enjoyed good health and stable relationships. He was brought by his mother to the hospital on 18th July 2010 for refusing to eat, which had led to severe weight loss and generalized weakness. His condition began seven months prior when his friends teased him about his plumpness and greedy appetite. Consequently, he started restricting his food intake, avoiding high-calorie foods and indulging routinely in extensive exercise. His parents tried to convince him to eat regular meals, but he refused. He was obsessed about his body shape and measured his waist and thigh circumferences regularly. His food intake decreased rapidly until his daily meal became no more than a cup of yogurt and pieces of cucumber. A few weeks before his visit to the hospital, he experienced severe fatigue, headaches, joints aches and attacks of epigastric pain followed by vomiting. He was treated by the local doctor with multivitamins, anti-emetics and anti-spasmodics, but without improvement.

The patient seemed to be overprotected by his mother. He was the youngest of seven siblings and a clever boy. She described him as being a graceful and obedient child, but somewhat of a perfectionist. There was neither a history of physical or mental illness nor sexual abuse during childhood.

On admission to the hospital, he appeared to be severely ill. He was pale, emaciated and dehydrated. He had lost about 20 kg during the past four months, according to his mother, who reported episodes of irritability and depressed mood with the decrease in weight. His body weight was 28 kg and height 147 cm, which is below the 2nd percentile for his age according to the growth chart. His BMI was 13.0, reflecting a 37% deficit in weight for his height [16].

Upon examination, he showed generalized muscle wasting, and his skin was dry and covered by lanugo hair. His chest was clear, and he had a scaphoid soft abdomen. His blood pressure was 90/60 mmHg, heart rate 55 bpm and body temperature 37.8°C. Laboratory tests were as follows: blood glucose: 60 mg/dl, blood urea: 18 mg/d, serum sodium: 136 mg/dl, serum creatinine: 0.6 mg/dl, serum cholesterol: 147 mg/dl, WBC: 2200, platelet count: 150000, ESR: 1, and HB: 12 mg/dl. Blood proteins were low with slightly elevated liver enzymes. The blood culture was negative. Thyroid functions tests and steroid hormones were normal. General urine examination and culture revealed an acute bacterial infection, which accounted for his fever. Skull, spine and chest x-ray, brain scan and abdominal ultrasound were all normal. A previous gastroscopy at another hospital showed mild mucosal atrophy. An ECG revealed sinus bradycardia. The patient was smaller and shorter than his matching peers. He looked attentive but indifferent to his serious condition. His speech was rational and did not reveal delusions or hallucinations. However, he expressed strong denial of his body appearance and insisted he had a normal body shape. His mood was depressed, but he denied suicidal ideation.

The patient was admitted on 18th July 2010 to Sulimania General Hospital and was referred later for psychiatric consultation. A liaison treatment plan was designed by the attending physician, psychiatrist and dietitian focusing on scheduled feeding under the dietitian's advice, which was to be achieved through a nasogastric tube. Caloric intake was measured to reach 2000 calories per day, and he was instructed to rest in bed under a nurse's supervision. This ameliorated his previous hypoglycemic attacks. He was prescribed oral cephalosporin 250 mg qds to treat his urinary infection. On the second day of his admission, the psychiatrist prescribed a 20 mg daily dose of fluoxetine to treat his depressed mood and food-related obsessions. A 5 mg nightly dose of olanzapine was prescribed to resolve the distorted thoughts about his body image and promote sedation. Nasogastric refeeding was continued for the first week in the hospital until oral feeding was established. The tube was removed on the 6th day. In addition, a few sessions of cognitive psychotherapy were conducted with the patient.

At the end of the third week, he became more realistic and rational about his body weight and was more compliant with eating normal meals. His mood and irritability improved. He was discharged after 24 days in the hospital, advised to abide by the regulations of the dietitian and continue his medication for three months. He was to report to the psychiatry clinic on alternate weeks. His body weight had increased to 30 kg.

He did not keep his appointments and appeared six months later at the clinic weighing 38 kg. He was enjoying better health, although he was still having eating peculiarities. He had maintained his daily exercise but was reluctant to gain more weight. After consultation, he was advised to continue on fluoxetine for another three months. The family was advised to escort him regularly for psychotherapy sessions and watch his dietary intake.

## Conclusions

The clinical presentation of anorexia nervosa among males is rare [2,5,6], with many subclinical cases being overlooked. Males and females tend to share similar clinical presentation and psychopathology except for amenorrhea. In addition, males are inclined towards strenuous exercise, have sexual concerns and show psychiatric comorbidity more often than females. Reports about age of onset in males vary. However, it is agreed that males tend to present at a later age than females probably because of the later onset of puberty and different social roles [5,6,17].

The influence of culture on the development of AN has long been appreciated and is believed to be more prevalent in industrialized and Western cultures, being far more common among young females than males and reflecting cross-cultural differences in the importance of thinness for women [7,8,17,18]. Apart from a few studies showing a propensity for anorexic-like behavior, the available literature indicates that anorexia nervosa is rare among females in the Arab culture [8-10,13,19]. Traditional values and cultural norms regards thinness as socially undesirable, with plumpness considered a sign of wellbeing in both genders and viewed as a symbol of fertility and womanhood in females [8,10,19,20]. A positive relationship between increased body weight and higher social class has been observed in the Arab culture, contrary to Western ideals [8,10,13,19,20]. It has been suggested in the available literature that exposure to Western values regarding body shape and weight can be blamed for the occurrence of anorexia nervosa in the Arab region [1,9,10,14,20].

Iraq, like many other Arab countries, is known for using English as the teaching medium, thus facilitating access to Western culture through satellite television, the Internet and periodicals. It is believed that exposure to the differences between the two cultures contributes to the etiology of eating disorders [21-23]. Through recent globalization, Western cultural norms have infiltrated many Arab societies and changed local traditional values regarding ideal body shape and weight. MR was a boy from an educated middle class family residing in a suburban area north of Iraq where local values did not favor thinness. There was easy access to media, including the Internet, which might have contributed to an internal conflict regarding body image.

We believe that nutritional correction by oral and nasogastric feeding helped in weight restoration of this patient, which is a prerequisite for the effective use of psychotropic interventions [24]. Using an oral antidepressant (flouxetine, 20 mg) improved the patient's gloom and irritability. The oral antipsychotic (olanzaine, 5 mg) ameliorated his weight-related beliefs and probably helped him gain weight by the end of the second week with no serious side effects apart from daytime sleepiness. Medicating anorexic patients with a combination of an antidepressant and antipsychotic has previously been tried and shown to be successful [10,19].

The patient was diagnosed with anorexia nervosa, according to DSM-IV diagnostic criteria and the Eating Disorder Inventory (EDI). His history and personality profile together with the nurturing attitude of his mother were strikingly similar to many previous reports in Western and Arab societies [25,26]. Despite the short period of hospitalization, lack of a special unit for eating disorders and brief psychotherapy, our patient showed significant improvement as a result of the available medical and psychiatric care he received.

## Consent statement

Written informed consent was obtained from the patient for publication of this case report and accompanying images. A copy of the written consent is available for review by the Editor-in-Chief of this journal.

## Competing interests

The authors declare that they have no competing interests.

## Authors' contributions

MSY and LDA contributed equally in editing and reviewing the manuscript. The final version has been read and approved by both authors.

**Figure 1:**
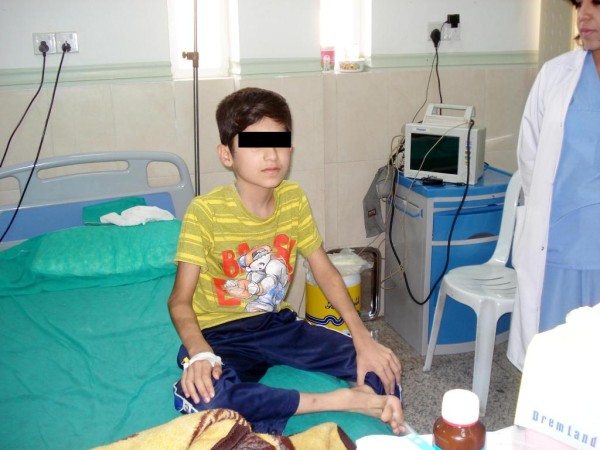
**The patient at admission to the hospital medical ward in July 2010**.

**Figure 2:**
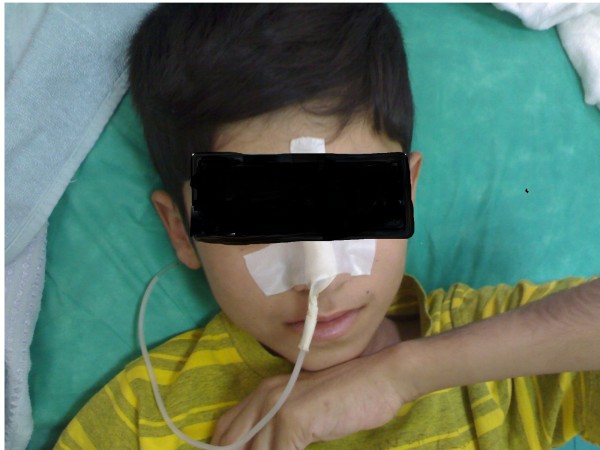
**The patient with a nasogastric tube 2 days after admission**.
